# Dissecting the Puzzling Roles of FAM46C: A Multifaceted Pan-Cancer Tumour Suppressor with Increasing Clinical Relevance

**DOI:** 10.3390/cancers16091706

**Published:** 2024-04-27

**Authors:** Giancarlo Lai, Federica De Grossi, Ilaria Catusi, Elisa Pesce, Nicola Manfrini

**Affiliations:** 1 INGM, Istituto Nazionale Genetica Molecolare Romeo ed Enrica Invernizzi, 20122 Milan, Italy; lai@ingm.org (G.L.); degrossi@ingm.org (F.D.G.); pesce@ingm.org (E.P.); 2 Department of Biosciences, University of Milan, 20133 Milan, Italy; 3 SC Clinical Pathology, SS Medical Genetics Laboratory, Fondazione IRCCS Cà Granda Ospedale Maggiore Policlinico, 20122 Milan, Italy; ilaria.catusi@policlinico.mi.it; 4 Department of Clinical Sciences and Community Health, University of Milan, 20122 Milan, Italy

**Keywords:** TENT5C, poly(A) polymerase, autophagy, PLK4, tumour suppressor, multiple myeloma

## Abstract

**Simple Summary:**

FAM46C is a tumour suppressor protein originally characterized in multiple myeloma. Recent findings, however, suggest a much broader involvement of FAM46C in cancer, underlining the clinical importance of fully dissecting the intracellular pathways it modulates. To date, FAM46C mechanistic role is still disputed and studies regarding its mode of action are sometimes contradictory, urging the need to summarize and clarify the results obtained so far. Here, by focusing primarily on the intracellular pathways modulated by FAM46C, the currently accepted models regarding its mode of action, the regulators of its expression and how it associates with increased/decreased cell sensitivity to anticancer agents, we provide the first comprehensive review on FAM46C.

**Abstract:**

FAM46C is a well-established tumour suppressor with a role that is not completely defined or universally accepted. Although FAM46C expression is down-modulated in several tumours, significant mutations in the *FAM46C* gene are only found in multiple myeloma (MM). Consequently, its tumour suppressor activity has primarily been studied in the MM context. However, emerging evidence suggests that FAM46C is involved also in other cancer types, namely colorectal, prostate and gastric cancer and squamous cell and hepatocellular carcinoma, where FAM46C expression was found to be significantly reduced in tumoural versus non-tumoural tissues and where FAM46C was shown to possess anti-proliferative properties. Accordingly, FAM46C was recently proposed to function as a pan-cancer prognostic marker, bringing FAM46C under the spotlight and attracting growing interest from the scientific community in the pathways modulated by FAM46C and in its mechanistic activity. Here, we will provide the first comprehensive review regarding FAM46C by covering (1) the intracellular pathways regulated by FAM46C, namely the MAPK/ERK, PI3K/AKT, β-catenin and TGF-β/SMAD pathways; (2) the models regarding its mode of action, specifically the poly(A) polymerase, intracellular trafficking modulator and inhibitor of centriole duplication models, focusing on connections and interdependencies; (3) the regulation of FAM46C expression in different environments by interferons, IL-4, TLR engagement or transcriptional modulators; and, lastly, (4) how FAM46C expression levels associate with increased/decreased tumour cell sensitivity to anticancer agents, such as bortezomib, dexamethasone, lenalidomide, pomalidomide, doxorubicin, melphalan, SK1-I, docetaxel and norcantharidin.

## 1. Introduction

In order to implement cancer patient therapy, novel targetable pathways are required. FAM46C is a recently-defined tumour suppressor which regulates numerous intracellular processes, but whose mode of action is still under investigation. Better defining how FAM46C functions and how it is modulating intracellular pathways will be fundamental for defining novel druggable targets.

Originally, the FAM46C protein was associated with viral responses. Specifically, the first-ever mention of FAM46C came from a screening to define novel interferon (IFN)-stimulated genes (ISG)s [1]. However, its role in modulating viral replication was not thoroughly explored, possibly due to controversial results regarding its effects [2,3]. Only recently is the association of FAM46C with viral infection starting to re-gain attention [4].

The true interest of the scientific community for FAM46C arose when genetic evidence revealed the gene to be significantly and exclusively mutated in MM patient samples [5,6] and by the subsequent studies demonstrating that FAM46C functions as a tumour suppressor [7,8].

The first functional studies were mostly performed in MM, but later, investigations also involved other cancer types, namely colorectal [9], prostate [10] and gastric cancer [11], and squamous cell [12] and hepatocellular carcinoma [7]. In these scenarios, FAM46C was shown to behave as a tumour suppressor modulating different intracellular pathways important for cell proliferation and survival, namely the MAPK/ERK [7], PI3K/AKT [13], WNT/β-catenin [11] and SMAD [14] pathways. Accordingly, FAM46C is now considered to be a potential pan-cancer prognostic marker [15].

Given the growing importance of FAM46C as a broad tumour suppressor, several groups have attempted to define the synergistic effects between FAM46C expression or inactivation and cancer cell sensitivity to anticancer drugs, sometimes obtaining contradictory results.

Outside of the tumoural environment, FAM46C was shown to be involved, by different means, in specific cellular developmental/differentiation processes, namely spermatogenesis [16] and osteoblast [17] and macrophage differentiation [18,19], making it of fundamental relevance to try to define its mechanistic mode of action.

Through the years, different models have been proposed in order to explain both FAM46C tumour suppressor phenotypes and its physiological role: the poly(A) polymerase model [20], in which FAM46C is proposed to polyadenylate the poly(A) tails of specific transcripts, stabilizing them; the PLK4 regulator model [9], in which, by inhibiting PLK4 activity, FAM46C is envisaged to restrict centriole over-duplication; and the intracellular trafficking model [21], in which FAM46C is proposed to function as a master regulator of intracellular trafficking dynamics. Each and every one of these models is fundamental in acquiring a general idea of the FAM46C mode of action, as no one model, on its own, can fully explain FAM46C-induced phenotypes.

Here, by describing and commenting on (1) the tumours in which the protein is actually functioning as a tumour suppressor, (2) the pathways correlated with FAM46C expression, (3) the models proposed for its mode of action, (4) how expression of FAM46C is regulated and (5) how the presence or absence of FAM46C affects cell sensitivity to anti-cancer drugs, we try to bring order to the heterogeneous literature regarding FAM46C.

## 2. The *FAM46C* Gene and Its Mutations in Cancer

*FAM46C* is part of the highly conserved *FAM46* gene family, which comprises at least one member in all animal phyla [22].

In almost all sequenced vertebrata, the *FAM46* family is composed of four members: *FAM46A*, *FAM46B*, *FAM46C*, *FAM46D*, each derived from duplication events of a common ancestor gene.

The four paralogs have different tissue expression, with *FAM46A*, *FAM46B* and *FAM46C* being potentially expressed in 81,18 and 66 tissues or cell types, respectively, and *FAM46D* being exclusively expressed in sperm cells [22]. Despite their differential tissue expression, all FAM46 proteins seem to be involved in different but fundamental biological processes. FAM46A was shown to be involved in retinal homeostasis [23] and in the maturation of Type I muscle fibres [24]. Both FAM46A and FAM46C were found to be important for proper bone formation [17,25,26] and macrophage activation [27]. FAM46B was shown to be essential for embryonic stem cell viability [28] and was associated with lupus nephritis [29]. FAM46C alone was shown to be involved in B cell maturation [20,30], while both FAM46C and FAM46D were shown to be involved in sperm cell differentiation [16,31]. All FAM46 proteins are also involved in cancer, with FAM46A being found associated with non-small cell lung cancer (NSCLC) [32], glioma [33], oesophageal [34] and ovarian carcinoma [35], FAM46B with prostate cancer [36,37] and NSCLC [38] and FAM46D with gastric cancer [39]. However, FAM46C stands out as the most studied in the tumoural environment. This is likely due to the fact that, aside from FAM46D, which is mutated in gastric cancer [39], FAM46C is the only family member consistently found altered in cancer patient cohorts [40,41,42]. Therefore, it was initially considered to have the highest potential therapeutic relevance. Specifically, FAM46C is exclusively mutated in MM, and as a result, most studies regarding its mode of action were originally conducted in the MM environment.

The *FAM46C* gene is located at chromosome 1, specifically at the 1p12 arm, and is organized into two exons and one intron, with its ORF being made up by 391 codons localizing exclusively on exon 2. Importantly, the *FAM46C* gene falls in a region of enhancer/super-enhancer sequences which are important for gene transcription upon B cell differentiation [43,44].

The *FAM46C* gene was originally identified as mutated in 13% of the cases analysed by Chapman and colleagues in their 2011 parallel sequencing of 38 MM patient genomes/exomes [5]. Later, similar mutation frequencies were confirmed by others using techniques ranging from fluorescence in situ hybridization (FISH) approaches to gene-targeted next generation sequencing (NGS) [45,46,47,48,49,50,51,52,53]. Mutation mapping revealed alterations throughout the entire ORF of *FAM46C*, with an exception in a small portion of the N-terminal region, with most mutations being either indels or missense single nucleotide variations predicted to have a deleterious effect on protein structure/functionality [54], suggesting that *FAM46C* behaves as a tumour suppressor gene.

Besides being highly mutated, *FAM46C* is also frequently deleted in MM patients. Boyd and colleagues were the first to find that the homozygous deletion of the 1p12 arm containing *FAM46C* occurred at high frequency in MM patients (19% of analysed cases) [6]. Such results were later confirmed by others [44,55]. Specifically, it was found that deletions of the 1p12 chromosome, containing *FAM46C* super-enhancer, translocated with the 8q24 locus, which comprises oncogenic *MYC*, in turn triggering *MYC* overexpression [43,44,56] and *FAM46C* inactivation.

Despite *FAM46C* being originally found to be significantly mutated only in MM, studies underlined the existence of alterations of the *FAM46C* gene sequence also in other cancers, i.e., gastric cancer (GC) and chromophobe renal cell carcinoma (CRCC) [57,58]. While, in five GC cell lines, two specific *FAM46C* point mutations (namely, c. 87C > G and c. 483C > T) were shown to correlate with decreased FAM46C expression levels, and low FAM46C levels served as a predictor of hepatic recurrence in patients with resectable GC [57], in CRCC the only mutation found in a cohort of 10 patients was predicted to be non-pathogenic [58]. While, in the first study, gene-targeted sequencing was performed on cell lines mostly derived from carcinomas, in the second analysis, NGS was performed on tumour samples at different clinical stages, making the two approaches drastically different but suggesting that *FAM46C* pathogenic mutations might indeed correlate with increased disease severity. In general, future analyses on more broad and specific cohorts are mandatory in order to define the relevance of *FAM46C* gene alterations in cancers other than MM.

## 3. FAM46C: A Multi-Cancer Tumour Suppressor

Through the years, FAM46C was found to be involved in several types of malignancies besides MM, namely colorectal, prostate and gastric cancer, squamous cell, hepatocellular and chromophobe renal cell carcinoma (Table 1). Furthermore, recently, FAM46C was established as being a pan-cancer prognosis factor important for predicting immunotherapeutic efficacy [15,59].

Below, we describe the effects of FAM46C modulation in different cancer types.

### 3.1. Multiple Myeloma

The first speculation that FAM46C could have a tumour suppressor role in MM came from the 2011 study by Boyd and colleagues, which found that deletion of 1p12 correlated with impaired overall survival (OS) of MM patients receiving autonomous stem cell transplantation [6]. It then took other studies [43,44] to confirm that the oncogenic effect of the 1p12 deletion was associated with the *FAM46C* gene and three more years to have the first confirmation that FAM46C was an actual MM tumour suppressor, in a study by Zhu and colleagues [8]. The authors not only demonstrated that wt FAM46C overexpression inhibited proliferation and induced apoptosis in different MM cell lines, but also showed that the FAM46C alterations found in MM patients abrogated all of these effects, indicating a survival advantage conferred by the FAM46C mutant phenotype [8]. Subsequent studies by different groups confirmed these results. Mroczek and colleagues demonstrated that, in MM cells, re-expression of wt FAM46C, but not of a loss-of-function allele, induced cell death and that FAM46C silencing favoured proliferation [20]; Herrero and colleagues demonstrated that either FAM46C knockout or down-modulation favoured MM cell invasion and migration [30]; and Kanasugi et al. demonstrated that knockout of FAM46C inhibited apoptosis and favoured cell cycle progression of MM cells [13]. The authors also found that knockout of FAM46C favoured MM tumour growth in a xenograft mice model [13], a result that was later confirmed by experiments in our lab, which showed that expression of wt FAM46C inhibited tumour growth and tumour cell proliferation [21], fully establishing FAM46C as a MM tumour suppressor.

### 3.2. Colorectal Cancer

Originally, evidence of a role for FAM46C in colorectal cancer (CRC) came from a seminal work published in 2020 by Kazazian and colleagues [9].

The authors found that FAM46C expression (1) was reduced in CRC tumour samples compared to adjacent normal mucosae and (2) decreased with CRC clinical stage progression. Recently, the Shi group not only confirmed these results but also proposed FAM46C to serve as an independent prognostic biomarker for CRC, with its reduced expression being associated with unfavourable prognosis [60], overall establishing a clear link between FAM46C and CRC.

### 3.3. Squamous Cell Carcinoma

FAM46C was found to be involved also in squamous cell carcinoma (SCC).

Three studies correlated FAM46C expression with this type of tumour, specifically with oral SCC, SCC of the lung and oesophageal SCC.

Xiaohua and colleagues were the first to describe FAM46C as a tumour suppressor in SCC. They used oral SCC cell lines with low or high FAM46C levels and either re-expressed or down-modulated the protein. They found a drastic reduction in proliferation and significant induction in apoptosis when FAM46C was re-expressed and diametrically opposing effects when FAM46C was down-modulated [12].

Xia and colleagues instead encountered FAM46C while performing an onco-miRNA screening on samples of SCC of the lung. They found that three miRNAs correlated with unfavourable prognosis, and all three had one common target: FAM46C. The authors suggest that the three miRNAs promote cell proliferation by inhibiting FAM46C expression, but only indirectly correlate FAM46C decrease with proliferation of lung SC cells [61].

Similarly, Ma et al. [62] originally encountered FAM46C as an onco-miRNA target in oesophageal SCC. Their demonstration of FAM46C’s tumour suppressor role was, however, more direct. They showed that FAM46C overexpression triggers apoptosis and negatively affects both the migratory and invasive capacities of human oesophageal cancer cells [62].

Overall, FAM46C can be envisaged as strong tumour suppressor in SCC.

### 3.4. Prostate Cancer

In 2020, a comprehensive work by Ma and colleagues demonstrated for the first time the involvement of FAM46C with prostate cancer (PC) [10]. The authors, at first, showed that FAM46C levels were reduced in PC tissues compared to noncancerous counterparts and subsequently described a clear anti-correlation between FAM46C expression and tumour aggressiveness. Accordingly, they also found that PC patients with high FAM46C expression levels had better overall survival compared to patients with low FAM46C expression and hence envisaged FAM46C abundance as a prognostic factor for PC patients.

Then, the authors shifted to in vitro models and demonstrated that FAM46C down-modulation increased proliferation, cell cycle progression and colony-forming capacity of PC cells while decreasing apoptosis.

Accordingly, opposing results were obtained when FAM46C was overexpressed. To further strengthen their results, the authors also generated a xenograft mice model and found that FAM46C overexpression drastically inhibited prostate tumour growth, fully demonstrating that FAM46C acts as tumour suppressor also in PC.

### 3.5. Hepatocellular Carcinoma

FAM46C role in hepatocellular carcinoma (HCC) has been demonstrated by two related works published in 2017 by the Xin lab [7,14].

The authors came across FAM46C while trying to determine the mode of action of the antimetastatic drug norcantharidin (NCTD) (see below). Since FAM46C expression was upregulated upon NCTD treatment in HCC cells, the authors assessed directly the effects of FAM46C upregulation/down-modulation both in HCC cell line models and in an in vivo mice model.

They found that FAM46C overexpression in HCC cell lines (1) inhibited proliferation, (2) triggered G2/M cell cycle phase arrest, (3) induced cell apoptosis and (4) inhibited both migration and invasion. Accordingly, FAM46C down-modulation had the opposite effects [7,14]. Results were also confirmed when the authors tested FAM46C tumour suppressor activities in vivo, as they found attenuated HCC formation in mice overexpressing FAM46C [7].

All together, the results obtained by the Xin lab clearly establish FAM46C as a HCC tumour suppressor.

### 3.6. Gastric Cancer

In 2017, a study by Tanaka and colleagues first presented FAM46C to the gastric cancer (GC) research community [57]. In their work, the authors performed a global mRNA expression profiling on tissues derived from GC patients with synchronous liver-confined metastasis and found that low FAM46C expression levels were significantly associated with larger GC tumour sizes. Moreover, patients with low FAM46C tumour expression were shown to have shorter disease-free survival, and reduced FAM46C levels were identified as an independent risk factor for recurrence.

All the prerequisites to define FAM46C as a tumour suppressor were in place; what was missing was a formal demonstration. Such a demonstration came in 2020 through a work published by Shi and colleagues [11]. The authors initially confirmed that FAM46C levels were lower in GC tumour tissues compared to non-tumoural counterparts. Next, they showed that FAM46C overexpression inhibited proliferation and cell cycle progression, but induced apoptosis in GC cells. When they down-modulated FAM46C, they found exactly the opposite results, formally demonstrating that FAM46C is a GC tumour suppressor.

### 3.7. Lung Cancer

FAM46C involvement in lung cancer (LC) is, to date, only a speculation, as a formal demonstration of an oncosuppressor role of FAM46C in this tumour type is missing. With this said, two convincing studies hypothesized FAM46C involvement in this type of disease and hence are worthy of being mentioned.

Apart from the previously described study by Xia and colleagues, in which the authors suggested FAM46C to be the downstream target of three different lung SCC-specific onco-miRNAs and hence responsible for inhibiting proliferation of cells derived from SCC of the lung [61], Li et al. hypothesized FAM46C to be a major player in LC progression. Specifically, they proposed FAM46C to be one of the downstream targets responsible for the tumour suppressor phenotypes induced by hypermethylated in cancer 1 (HIC1) protein in LC cells [63], namely, inhibition of cell migration and colony formation.

Despite the clear phenotypes described, future studies are mandatory to fully establish FAM46C as a LC tumour suppressor.

Overall, the general idea we get is that FAM46C behaves as a bona fide pan-cancer tumour suppressor, exerting a negative regulation on crucial cellular processes such as cell cycle progression, proliferation, migration, and invasion, while promoting apoptosis. Moreover, FAM46C levels in patients seem to be generally anti-correlated with disease severity, making its expression level a promising valuable feature for patient stratification.

## 4. Signalling Pathways Regulated by FAM46C

In line with its tumour suppressor role, FAM46C expression was shown to anti-correlate with activation of pathways required for cell cycle entry and cell proliferation/survival, namely, the MAPK/ERK, PI3K/AKT, WNT/β-catenin and SMAD pathways (Table 2). Here, we will summarize, for each pathway, the evidence regarding FAM46C involvement.

### 4.1. MAPK/ERK Signalling

Anti-correlation between FAM46C expression and activation of the MAPK/ERK signalling pathway was found in MM, HCC and oral SCC.

Originally the first to connect FAM46C expression with inactivation of ERK signalling were Zhang and colleagues [7], who demonstrated that FAM46C overexpression in HCC cells reduced RAS protein levels and, concomitantly, also the phosphorylation of both MEK1/2 and ERK1/2 and the levels of downstream target Bcl-2. Accordingly, the authors showed that FAM46C knockdown caused the opposite effects. Such results were at least in part reproduced in MM cells by Zhu and colleagues [8], who demonstrated that FAM46C knockout actually triggered massive ERK phosphorylation and an increase in the protein levels of MEK/ERK downstream target Bcl-2.

In oral SCC, Xiaohua and colleagues instead went a bit further; they not only showed that FAM46C overexpression was correlated with decreased MEK1/2 and ERK1/2 phosphorylation, but also found that FAM46C-induced apoptosis could be at least in part inhibited by triggering the MEK/ERK pathway with growth factors [12]. Accordingly, on the contrary, they also showed that FAM46C down-modulation could, at least in part, suppress the apoptotic effect induced by ERK1/2 inhibition, further strengthening the FAM46C-MEK/ERK connection in cancer.

FAM46C was shown to regulate the MEK/ERK pathway also outside of tumoural environments, as in lipopolysaccharide (LPS)-stimulated cardiomyocytes its overexpression was again shown to specifically reduce phosphorylation of ERK1/2 [70].

All together, these results clearly envisage a tight regulation of FAM46C on activation of the MEK/ERK pathway.

### 4.2. PI3K/AKT/mTOR Pathway

Recently, FAM46C expression was shown to strongly anti-correlate with activation of the PI3K/AKT/mTOR pathway.

Kanasugi and colleagues originally established the FAM46C-PI3K/AKT connection both in vitro and in vivo in MM [13]. They found that MM cell lines lacking FAM46C actually had hyperactivation of the PI3K/AKT pathway, as demonstrated by a drastic increase in AKT phosphorylation, an effect that could be suppressed by FAM46C re-expression.

These results were in line with preliminary experiments performed by Herrero and colleagues [30], in which Wortmannin and LY2940002, two well-established PI3K inhibitors, were found to partially suppress the increased cell migration effect caused by FAM46C down-modulation in MM cells.

Similar results were obtained when tumours derived from FAM46C knockout MM cells were established in mice: loss of FAM46C induced phosphorylation not only of AKT but also of its downstream target FOxO1/3A [13].

Overall, comparable results were also obtained in prostate cancer by Ma and colleagues [10], which showed that FAM46C overexpression reduced AKT phosphorylation in the DU145 prostate cancer cell line model.

In both studies [10,13], FAM46C was also shown to positively regulate PTEN, the master negative regulator of the PI3K/AKT signalling pathway. However, while Kanasugi and colleagues found that FAM46C expression affected PTEN activity but not its protein levels [13], Ma and colleagues showed that FAM46C expression increased protein but not transcript levels of PTEN [10], suggesting a post-transcriptional regulation. The authors indeed showed that FAM46C stabilized the PTEN protein by inhibiting its ubiquitination.

Overall, these results establish a clear link between FAM46C expression and inhibition of the PI3K/AKT pathway.

### 4.3. WNT/β-Catenin Pathway

Among the different intracellular pathways regulated by FAM46C, Shi and colleagues envisaged also the canonical WNT pathway [11]. In their studies in GC, the authors were originally triggered by the anti-correlation, in patient-derived tissues, between FAM46C expression and WNT signalling, a result that was subsequently confirmed when they found anti-correlation also between expression of FAM46C and that of β-catenin both in GC tissues and cell lines. Based on this strong evidence, the authors then formally demonstrated that all FAM46C-induced phenotypes in GC, namely inhibition of proliferation, of cell cycle progression and induction of apoptosis, were actually dependent on inhibition of WNT/β-catenin signalling. They did so by treating either FAM46C-expressing cells with LiCl, an agonist of WNT/β-catenin, or FAM46C down-modulated cells with DKK1, a WNT/β-catenin inhibitor, and finding, in the first case a suppression and in the second case a restoration of FAM46C-induced phenotypes.

Despite this study giving us the first clear indication of a connection between FAM46C and the canonical WNT pathway, further studies are necessary, firstly, to confirm these results, and secondly, to check for involvement of the WNT/β-catenin pathway on FAM46C-induced phenotypes also in other cancers.

### 4.4. TGF-β/SMAD Pathway

The involvement of FAM46C with the TGF-β pathway was observed by Wan and colleagues in HCC cell line models [14]. While trying to determine the anti-metastatic effects of FAM46C in HCC cells, the authors found that FAM46C expression and down-modulation were inhibiting or favouring, respectively, the phosphorylation of SMAD2/3, a downstream target of TGF-β. By showing that SMAD 2/3 phosphorylation correlated with HCC metastatization, the authors proposed a model in which FAM46C exerts its anti-metastatic function by negatively regulating the TGF-β/SMAD pathway.

To date, this is the only paper published connecting FAM46C with the TGF-β/SMAD pathway. Hence, further studies are mandatory to confirm this connection and extend it to other cancers.

### 4.5. The FAM46C-MYC Connection

Despite being broad and sometimes functionally distant from one another, the intracellular pathways inhibited by FAM46C all have in common one downstream target: transcriptional factor MYC. This suggests that FAM46C-induced intracellular pathway modulation might indeed be aimed at ultimately downregulating MYC expression.

In line with this idea, numerous studies regarding FAM46C have indeed found anti-correlation between FAM46C and MYC expression [8,11,61], suggesting that the tumour suppressor effect of FAM46C relies, at least in part, on inhibiting MYC.

To further strengthen the FAM46C-MYC connection stand the previously discussed rearrangements, frequently found in MM patients, which occur between the FAM46C super-enhancer locus and the MYC promoter (see the “The *FAM46C* gene and its mutations in cancer” section), which have the opposite effect of hyperactivating MYC through FAM46C inactivation.

MYC is one of the most common and well-studied pan-cancer oncogenes, while FAM46C is now becoming a well-established pan-cancer tumour suppressor, making this anti-correlation really attractive and exploitable for future therapy implementation.

## 5. FAM46C Functional Models

**Figure 1:**
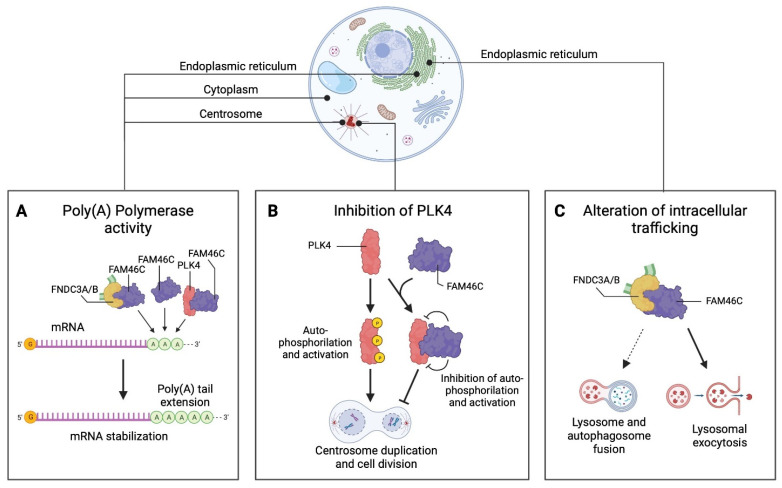
Proposed FAM46C functional models. (**A**) Poly(A) polymerase model, (**B**) PLK4 inhibitor model and (**C**) regulator of intracellular trafficking dynamics model. Image created with BioRender, https://www.biorender.com/ accessed on 15 April 2024.

In general, so far, three complementary models regarding FAM46C function have been proposed: (1) the non-canonical poly(A) polymerase model, (2) the PLK4 regulator/inhibitor model, and (3) the intracellular trafficking modulator models (Figure 1 and Table 3). Here, we will present them, discussing how they actually integrate and synergize with one another.

### 5.1. FAM46C as a Poly(A) Polymerase

The first, and most accepted, model proposed for FAM46C function is the poly(A) polymerase model (Figure 1A), in which not only FAM46C but all FAM46 family members are envisaged as poly(A) polymerases capable of modifying the poly(A) tails of specific transcripts, in turn stabilizing them and favouring their expression.

That FAM46C could function as a poly(A) polymerase was originally proposed by Kuchta and colleagues in 2016 [22]. By integrating different bioinformatics approaches, the authors categorized FAM46 proteins as non-canonical poly(A) polymerases, possibly actively modifying RNA 3’ ends. Specifically, through sequence-to-structure alignment, they identified the putative nucleotidyl transferase (NTase) domain, important for enzyme activity, and the putative PAP/OAS1 domain, important for nucleotide triphosphate binding, in all FAM46 family proteins.

Such in silico predictions were first confirmed by Mroczek and colleagues [20], by performing both in vitro polyadenylation and RNA-tethering assays. Despite the activity of FAM46C in the polyadenylation assay being weak, a result later confirmed by our group and others [21,28,64,65], the authors showed that by tethering FAM46C to a reporter mRNA, the protein was indeed capable of polyadenylating its target sufficiently to stabilize it and to enhance its expression. Accordingly, the authors then found the endogenous targets stabilized by FAM46C in MM, namely, ER-targeted transcripts, and proposed that, by stabilizing ER-targeted transcripts and hence favouring their translation, FAM46C triggers unmanageable ER stress and consequent cell death of already ER-stressed MM cells. The capability of FAM46C to stabilize specific transcripts, and hence favour their expression, was later confirmed by others both in MM [30,66] and outside the tumoural environment, specifically in plasma cells [30], terminally differentiated B cells [71] and osteoblasts [17], making the poly(A) polymerase model the one most widely accepted to explain a mechanistic role of FAM46C.

However, a few points regarding such poly(A) polymerase activity still require elucidation.

Although it is clear that in different cell types, which possess completely different functions, i.e., B cells and osteoblasts, FAM46C stabilizes different ER-targeted transcripts [17,20], it is less clear why in different MM cell lines FAM46C seems to have drastically different effects on transcript stabilization, ranging from hundreds of upregulated mRNAs upon FAM46C overexpression, to only a few transcripts significantly deregulated upon re-expression or down-modulation [20,21,30], suggesting that either FAM46C has a different behaviour in different MM cell types or simply that different experimental approaches might have had differentially estimated FAM46C stabilization capabilities, making the results not fully comparable.

Since, to date, what sequences are recognized by FAM46C and which auxiliary factors are required for target binding/identification is still an open issue, it is complicated to draw any final conclusion in this regard.

Recently, however, Liu and colleagues demonstrated that the poly(A) activity of FAM46 proteins is inhibited by the alpha isoform of the BCCIP protein [72], suggesting that modulators of FAM46C Poly(A) activity might indeed enter the picture.

One more concern comes from the fact that the poly(A) activity does not fully explain the tumour suppressor phenotypes induced by FAM46C. For example, in MM cells, the reduced poly(A) activity of different FAM46C mutants does not always correlate with impaired tumour suppressor phenotypes [65], and mutants predicted not to affect FAM46C poly(A) activity actually suppress FAM46C-induced phenotypes [21], suggesting that FAM46C poly(A) activity itself is not sufficient to fully explain its tumour suppressor effects.

To further complicate the picture, despite the poly(A) activity of FAM46C was shown to stabilize its target transcripts in most cell types, this does not seem to be the case for macrophages, where FAM46C poly(A) activity was shown to increase transcript translational efficiency but not transcript stability [27], making it difficult to give a final explanation about the functional role of FAM46C-induced polyadenylation.

### 5.2. FAM46C as an Inhibitor of PLK4

The second model connects FAM46C with control of centrosome duplication (Figure 1B).

FAM46C was shown to reside in the cytoplasm [20], at the ER [21,66] and, thanks to interaction with the master regulator of centrosome duplication PLK4 [73,74,75], also at the centrioles [9,64]. This latter localization was shown to be required by FAM46C to fully exert its tumour suppressor functions. Specifically, Kazazian and colleagues demonstrated an active role of FAM46C in regulating centrosome homeostasis [9]. They showed that FAM46C functions as a tumour suppressor by inhibiting centriole over-duplication, an event often seen in cancer cells, by blocking the autophosphorylation capacity of PLK4 in a way that is totally independent of its poly(A) polymerase activity (Figure 1B).

Intriguingly, they also showed that FAM46C is the only member of the FAM46 family to bind to PLK4 [9]. Being also the family member with the lowest poly(A) polymerase activity [20,65], it is tempting to speculate that, among the FAM46 proteins, FAM46C might have evolved toward a role which is more associated with non-poly(A) polymerase functions compared to the other family members. One of these functions could actually be the regulation of PLK4 functionality. Future studies will be required to validate this hypothesis and to establish to which extent the two functions of FAM46C cross-talk with one another. In line with this view, Zheng and colleagues found that FAM46C was essential, independent of its poly(A) activity, for fastening sperm cell and flagellum in mice by localizing at the manchette, a transient structure composed of microtubules [16].

Despite being clear and straightforward, this model is, however, still debated, since in their study Chen and colleagues confirmed the importance of FAM46C localization at the centriole but did not confirm its effect on centriole over-duplication [64].

### 5.3. FAM46C as a Regulator of Intracellular Trafficking Dynamics

The last model we will discuss was suggested by our lab [21] and proposes that, by localizing at the ER thanks to interaction with fibronectin type III domain containing 3A and 3B (FNDC3A and FNDC3B) [21,66], two proteins known to be involved in cell development [76,77], but also with disease [78,79], and specifically cancer [80,81,82,83,84,85], FAM46C regulates intracellular trafficking (Figure 1C). In our work, we found that expression of FAM46C in MM cell lines was coupled with only a really mild alteration of RNA levels and a really weak poly(A) activity, comparable to that of the loss-of-function FAM46C variant D90G. Instead, FAM46C expression triggered a significant alteration of intracellular vesicle transportation dynamics, with consequent deregulation of non-canonical protein secretion and autophagy, the latter effect causing accumulation of intracellular protein aggregates, an event that in already ER-stressed MM cells induced apoptosis, well explaining FAM46C tumour suppressor role.

The idea that FAM46C is involved in intracellular trafficking and that this function is not dependent on its poly(A) polymerase activity is in accordance with the previously mentioned work by Zheng and colleagues [16], in which FAM46C was shown to play a crucial role in sperm cell development by localizing at the manchette, a microtubule-based structure vital for nuclear shaping, protein trafficking, and overall spermatid movement.

Moreover, in agreement with this model, the cross-talk between FAM46C and vesicle dynamics, specifically autophagy, was confirmed by Fucci and colleagues, who showed that FAM46C can indeed interact with autophagosomal protein p62 [66], and by our lab in a later study, in which we demonstrated that FAM46C is capable of inhibiting lentiviral particle production in HEK 293T cells by negatively regulating autophagy [21].

However, just like for the previously described models, the intracellular trafficking modulation model also requires further elucidation, specifically, in terms of defining which enzymatic activity of FAM46C, if any, is involved and which intracellular pathways, among those regulated by FAM46C, might affect or be affected by vesicle trafficking modulation.

### 5.4. The Comprehensive Model

Overall, it is clear that each of the above-mentioned functional models on its own cannot fully explain FAM46C mode of action. The only way to explain the plethora of FAM46C-induced phenotypes, both in tumoural and non-tumoural environments, is to consider the three activities described as coexisting and possibly cross-talking with one another. FAM46C can localize in the cytosol, at the ER and/or at the centriole and, based on the cellular location, it might preferentially function in one of the ways described. In every cellular location FAM46C can polyadenylate and stabilize its targets (Figure 1A). When specifically bound at the centrioles, FAM46C can also function in slowing down microtubule duplication by regulating PLK4 activity (Figure 1B), while when specifically bound at the ER, we can envisage FAM46C as being involved also in regulating vesicle trafficking (Figure 1C).

However, how these functional models are connected with the intracellular pathways modulated by FAM46C still requires investigation.

Is intracellular pathway inhibition dependent on FAM46C poly(A) activity? Is it dependent on differential trafficking dynamics? Or does it depend on both? Further studies are required to better dissect these issues.

## 6. Regulation of *FAM46C* Gene Expression

Outside of the tumoural environment, FAM46C was shown to be an important factor for the correct differentiation of several cell types. Specifically, FAM46C was found upregulated during plasma cell [30,66,71] and sperm cell differentiation [16] and, along with FAM46A, during macrophage [18,19,27] and osteoblast differentiation [17].

Moreover, we recently demonstrated that *FAM46C* is a type I and type II IFN-stimulated gene, important for blocking lentiviral replication [4], overall suggesting that FAM46C expression is important in different cellular scenarios and, hence, must be tightly regulated.

Here, we will concisely summarize what is known regarding modulation of FAM46C expression, considering both positive and negative regulators (Table 4).

### 6.1. Positive Regulators of FAM46C Expression

#### 6.1.1. IFN-α and -γ

Early studies correlated FAM46C expression with IFN responses [1], but only recently has our lab actually demonstrated that FAM46C expression is induced upon type I and type II IFN administration. Specifically, we showed that this is true for both IFN-α and IFN-γ in macrophages, CD4^+^ T and dendritic cells [4]. However, unlike common ISGs, whose expression increases up to 1000-fold upon IFN administration, we found that FAM46C is upregulated only 2- to 4-fold, based on the stimuli and cell type analysed, suggesting it to be an overall weak ISG. Further studies will be fundamental to define if FAM46C is regulated also by other types of IFNs and which transcriptional factors regulate its expression downstream of IFN signalling.

#### 6.1.2. IL-4 and TLR Receptor Activators

In line with FAM46C involvement in plasma-cell differentiation, Bilska and colleagues found that FAM46C expression is specific to the later stages of B cell lineage differentiation [71]. The authors specifically showed that FAM46C expression in B cells occurred upon concomitant administration of LPS and IL-4 [71] but, more generally, also by innate signalling via specific toll-like receptors (TLRs), namely by engagement of either TLR1/2, TLR2, TLR4, TLR6/2 or TLR9. These results, besides demonstrating that FAM46C expression is triggered in B cells, introduce the tempting idea that FAM46C might also be broadly involved in global innate immunity.

#### 6.1.3. PRDM1 and HIC1

FAM46C expression was shown to be correlated with that of two transcriptional regulators, namely, transcriptional factor PRDM1 and transcriptional repressor HIC1. PRDM1 is the master regulator of plasma cell differentiation and is fundamental for steering immunoglobulin production [87], while HIC1 is a transcriptional repressor known to be frequently inactivated in cancer [63].

Fucci and colleagues, in their 2020 paper, showed that down-modulation of PRDM1 caused a drastic reduction in FAM46C protein levels in MM cells [66], while Li and colleagues instead found that loss of HIC1 correlated with decreased FAM46C levels in LC [63], suggesting that HIC1 might positively regulate, possibly indirectly, transcription of FAM46C.

Despite this evidence, the connection between the regulation of FAM46C expression by these transcriptional modulators and upstream signalling still requires elucidation.

### 6.2. Negative Regulators of FAM46C Expression

So far, negative regulation of FAM46C expression has been shown to occur mainly by targeting FAM46C transcript stability. Several microRNAs (miRNAs) were shown to be involved in destabilization of FAM46C transcripts, and their activity was shown to be regulated by different competing endogenous RNAs (ceRNAs), including both long non-coding (lnc) and circular (circ) RNAs.

In the tumoural environment, FAM46C was shown to be a target of miR10-b in osteosarcoma [67], of hsa-miR-1269a in oesophageal SCC [62] and of miR-296-5p, miR-324-3p, and miR-3928-3p in SCC of the lung [61].

In a high-throughput screening for defining novel MM-specific lncRNAs, Lu and colleagues found that expression of MSTRG.13132 correlated with that of FAM46C [86]. However, more direct experimental studies are required to confirm if, and define how, FAM46C expression is being regulated by MSTRG.13132.

Outside of the tumoural environment, FAM46C levels were shown to be important for differential polarization of macrophages. Specifically, FAM46C overexpression correlated with differentiation towards the M2 phenotype [19], while its down-modulation was shown to be required for differentiation towards the M1 subtype [18]. In this scenario, FAM46C levels were shown to be tightly regulated through miRNA targeting, namely by miR-657 and miR-4668-5p. Expression of miR-657 was shown to decrease FAM46C transcript and protein abundance, causing increased proliferation and migration and specific polarization to the M1 phenotype [18], while the sponging of miR-4668-5p by circRNA-17725 was shown to favour FAM46C transcript stability with concomitant induction of the M2 phenotype [19].

Overall, the idea we get is that FAM46C expression is regulated at different levels and by different means in different cellular subtypes, further underlining the relevance of FAM46C both in and out of the tumoural environment.

Despite its exact mechanistic role not being clear, what is clear is that FAM46C behaves as an overall regulator of cell proliferation. Specifically, by inhibiting proliferation in tumoural contexts, FAM46C behaves as a tumour suppressor, while by regulating proliferation in physiological contexts, FAM46C functions as an important cell development regulator. This broad activity is indeed coherent with the fact that FAM46C expression can be triggered by several and different upstream signalling factors.

## 7. Impact of FAM46C Expression on the Effects of Anticancer Drugs

Despite the mode of action of FAM46C still requires elucidation, its involvement in cancer disease and disease-related pathways is undoubtable and should be exploited for therapy implementation.

Accordingly, among the several studies aimed at defining the role of FAM46C, several have tried to characterize how its (over)expression or depletion/knockout had implications on the efficacy of anticancer drugs. Intriguingly, FAM46C was found to impact anticancer agents in opposing ways: in some cases, it augmented drug therapeutic function, while in some others it dampened it. Overall, FAM46C lack or expression was shown to affect the clinical outcome or effect of a wide range of different anti-cancer drugs (Table 5), making it promising to focus on *FAM46C* gene alterations/expression levels for implementing patient therapy.

Below is a list of anti-cancer drugs, both MM-specific and non-MM-specific, and the relative effect of FAM46C expression/down-modulation on their efficacy.

### 7.1. MM

#### 7.1.1. Bortezomib

Being MM cells highly secreting plasma cells with high ER stress, one of the most widely used approaches for MM patient therapy is to target the proteasome. The most widely used proteasome inhibitor in MM is bortezomib (BTZ). How FAM46C expression affects cells’ sensitivity to BTZ was thoroughly assessed but has given contradictory results.

Originally, Zhu et al. observed that FAM46C knockout had no effect on BTZ treatment in OCI-MYC5 MM cells [8], a lack of effect that was later confirmed by Herrero and colleagues in RPMI-8226 cells [30].

Kanasugi and colleagues confirmed this result in KMS-11 MM cells but, intriguingly, found that FAM46C knockout increased BTZ sensitivity in the ANBL-6 cell line model [13], a result later confirmed in patient-derived bone marrow mononuclear cells (BMMC) by Zhang and colleagues [68]. Moreover, they also found that the combination of BTZ with either PF-04691502 or afuresertib, a PI3K inhibitor and AKT inhibitor, respectively, caused more sensitivity in FAM46C knockout KMS-11 and OCI-MY5 cells compared to BTZ alone.

To further complicate the picture, one recently published work showed that MM cells with low FAM46C levels, derived from a VQ mice model, were instead less sensitive to BTZ treatment compared to those with higher FAM46C levels [69].

These overall differential effects are probably due to the widely diverse experimental models and procedures used, possibly carrying intrinsic biases due to differential ER stress and FAM46C expression levels. However, they underline the involvement of FAM46C in BTZ responses and the importance of performing more focused drug-sensitivity studies in this direction.

A synergistic effect between FAM46C and BTZ administration would indeed be expected and easily explained by the ER stress signature correlated with FAM46C [21,66].

#### 7.1.2. Dexamethasone

Dexamethasone is a corticosteroid commonly used in cancer therapy to prevent the side effects caused by chemotherapy but is also used for its anti-inflammation, anti-angiogenesis effects.

Zhu and colleagues found that FAM46C overexpression caused sensitivity to dexamethasone in KMS-11, OPM-2 and RPMI-8226 MM cell lines and that its knockout instead caused resistance to the drug in both OCIMY5 and XG1 cell lines [8].

Accordingly, Zhang and colleagues also found a correlation between FAM46C expression and sensitivity to dexamethasone, as patient-derived BMMC with reduced FAM46C levels were less sensitive to the drug [68].

Such effects were, however, not recapitulated by Herrero et al., since when they tested drug sensitivity of RPMI-8226 MM cells harbouring either wt levels or lack of FAM46C they found no differences [30], calling into question an actual synergistic effect between FAM46C expression and sensitivity to dexamethasone.

Given the general anti-proliferative role of FAM46C and the anti-angiogenic properties of dexamethasone, we would indeed expect a synergy between FAM46C expression and dexamethasone administration. Further studies are indeed mandatory to elucidate this issue and to determine which intracellular pathways are involved.

#### 7.1.3. Lenalidomide and Pomalidomide

Lenalidomide and pomalidomide are thalidomide analogues with anti-angiogenic and immunomodulatory properties. While lenalidomide is used in frontline MM treatment, pomalidomide is usually used for treatment of lenalidomide-refractive MM.

Zhu and colleagues found that FAM46C overexpression caused sensitivity to lenalidomide in both OPM-2 and KMS-11 MM cell lines and that, accordingly, its knockout caused resistance to the drug in MM XG1 cells [8]. However, such results were not confirmed by Kanasugi et al., who found no effect of FAM46C knockout on lenalidomide sensitivity in either KMS-11 or AMBL-6 cell lines [13]. The authors found that FAM46C knockout ANBL-6 MM cells were instead more sensitive to pomalidomide treatment, a result that, despite not being recapitulated in KMS-11 cells, suggests a possible differential synergistic effect between FAM46C expression and lenalidomide or pomalidomide treatment.

Given that one of the effects of either lenalidomide or pomalidomide administration is down-modulation of MYC [88,89], one would indeed expect synergism between FAM46C expression and administration of either of the two. Further and more specific studies are indeed required to fully establish if FAM46C expression is synergistic or not with the anti-cancer activities of either lenalidomide or pomalidomide.

#### 7.1.4. Doxorubicin and Melphalan

Doxorubicin and melphalan are two of the most widely-used chemotherapeutics in MM.

Doxorubicin is an anthracyline drug usually used in combination with bortezomib for MM treatment but also utilized to treat leukaemias, non-Hodgkin lymphomas and a broad range of other cancers [90], while melphalan is an alkylating agent used for general treatment of haematological malignancies.

Kanasugi and colleagues showed that FAM46C knockout causes resistance to either doxorubicin or melphalan treatment in both KMS-11 and OCI-My5 MM cells [13]. Herrero and colleagues found instead no effect of FAM46C knockout on melphalan treatment in both RPMI-8226 and JJN3 MM cells [30], suggesting that FAM46C expression is possibly synergistic only with doxorubicin administration. Given that doxorubicin functions primarily by DNA intercalation and by generation of free radicals, this synergistic effect is in agreement with that described by Fucci and colleagues, who showed that FAM46C expression triggers ROS accumulation [66]. Future studies are, however, required to fully establish FAM46C involvement in doxorubicin responses.

#### 7.1.5. SK1-I

Sphingosine kinase (SK) inhibitors have been recently shown to be promising drug candidates for MM treatment [91], as confirmed by the fact that estramidol, a selective sphingosine 1-phosphate receptor modulator, and sonepcizumab, a sphingosine 1-phosphate antibody, are in a phase two clinical trial [92,93] and that opaganib, a specific SK-2 inhibitor, is in phase 1 clinical trial [94].

Recently, we have shown that, in both LP-1 and OPM-2 MM cells, overexpression of FAM46C, sensitized cells to treatment with SK1-I, an inhibitor of both SK1 and SK2. Moreover, down-modulation of FAM46C in those same cell lines decreased sensitivity to SK inhibition [21], suggesting a clear synergistic effect between FAM46C expression and blockade of the SK pathway. This synergism is not surprising, given the involvement of FAM46C in intracellular trafficking [21] and the relevance of SKs in modulating the metabolism of lipids, the major components of intracellular vesicle compartments. Other studies are, however, required to confirm these results in vitro and evaluate the synergistic effect of FAM46C expression and SK inhibition also in vivo.

### 7.2. Other cancers

Synergies between FAM46C expression levels and effectiveness of anti-cancer drug treatment have also been assessed outside of MM, namely in prostate cancer, HCC and CRC, focusing on two drugs: docetaxel and norcantharidin (NCTD).

#### 7.2.1. Docetaxel

Docetaxel is a well-established anti-mitotic chemotherapeutic used for the treatment of different cancer types, including breast, ovarian, non-small cell lung and prostate cancer.

Specifically, docetaxel has been a first-line cytotoxic treatment for prostate cancer for more than 15 years [95].

Ma et al. clearly established a role for FAM46C in docetaxel chemosensitivity in prostate cancer [10].

The authors showed that FAM46C overexpression makes DU145 prostate cancer cells more sensitive to docetaxel treatment and, accordingly, that FAM46C down-modulation in the 22RV1 prostate cancer cell model makes them less sensitive to docetaxel administration. Moreover, the authors confirmed their results in patient-derived xenograft mice models of prostate cancer [10], further strengthening the idea that FAM46C expression is synergic with docetaxel-induced cytotoxicity.

Docetaxel anti-mitotic role is exerted primarily by targeting microtubules [95] and by interfering with microtubule dynamics. Hence, FAM46C-induced PLK4-inhibition might be what synergises with docetaxel treatment.

#### 7.2.2. NCTD

NCTD is a structural derivative of cantharidin (CTD) and possesses anticancer properties, including inhibition of proliferation, induction of apoptosis and inhibition of migration and has been recently shown to affect a wide range of cancers, e.g., liver and gastric cancer [96].

FAM46C was shown to be significantly involved in NCTD mode of action in HCC cells.

Specifically, in two separate works, the Xin laboratory showed that the apoptotic, anti-proliferative and anti-metastatic effects induced by NCTD in the HCC SMMC-7721 and MHCC-97H cell lines relied, at least in part, on NCTD-dependent induction of FAM46C expression [7,14]. These results clearly pinpoint an involvement of FAM46C in NCTD-induced phenotypes.

Given the broad-cancer effect of NCTD, future studies will be required to establish an eventual effect of the NCTD-FAM46C axis also in other tumours.

Despite the opposing effects of FAM46C expression in combination with different drug treatments, the general idea for future studies would be that of specifically testing selected drugs which are known to target pathways regulated by FAM46C and determine their efficacy in the presence or absence of FAM46C.

It would be tempting to speculate that in the near future we might be capable of stratifying patients based on FAM46C expression levels and/or mutational status and define, a priori, the best therapeutic approach to be used.

## 8. Concluding Remarks

The need to define novel and more efficient therapeutic strategies to fight cancer has turned attention to the discovery of novel cancer-related genes and to the characterization of their mode of action.

In line with this idea, with this review, we tried to organize the extensive, and sometimes contradictory, literature regarding the novel pan-cancer FAM46C, providing an exhaustive and comprehensive picture about its crucial roles.

Independent of the tumoural setting and mutational burden of FAM46C, three models have been proposed to explain FAM46C-induced phenotypes: the poly(A) polymerase, the PLK4 inhibitor and the regulator of intracellular trafficking models.

As mentioned earlier, although the poly(A) polymerase model stands as the most widely accepted within the scientific community, it falls short of explaining the multitude of effects associated with FAM46C expression and needs to be implemented by the other two.

The general idea that emerges is that none of the three models discussed, on its own, can fully describe FAM46C’s mode of functioning, leading us to propose a comprehensive model in which FAM46C behaves differentially based on the different cellular compartment (Figure 1) and, possibly, on the different cell type considered.

It is tempting to speculate that, physiologically, in different cell lines, or pathologically, in different cancer types, FAM46C behaves “primarily” through one of the models proposed. So, in highly secreting B cells or myeloma cells, where Ig secretion is key, FAM46C might be mainly involved in transcript stabilization and in organization of intracellular trafficking dynamics, while in cells, like melanocytes, in which secretion is less relevant, FAM46C might mainly exert its function by modulating centriole structure and duplication, an event which is similarly recapitulated in spermatids, where FAM46C is involved in maintaining the correct structure of the microtubule-derived manchette.

What is intriguing is that none of the above-mentioned models was actually shown to explain FAM46C-dependent inhibition of the MAPK/ERK, PI3K/AKT, WNT/β-catenin and SMAD survival pathways.

The only explorations in this direction were performed to test if transcript polyadenylation could account for FAM46C-dependent inhibition of the PI3K/AKT axis through stabilization of the mRNA encoding for PI3K/Akt pathway inhibitor PTEN. Different studies demonstrated that this does not seem to be the case [10,13,65], and a mechanism other than poly(A) adenylation of PTEN was proposed, namely PTEN protein stabilization through inhibition of its poly-ubiquitination [10], underlining a strict connection between FAM46C and the proteasomal machinery. FAM46 proteins were indeed already shown to be involved in protein stability, as FAM46C antiviral activity was shown to depend on autophagic dampening, an event which in turn was proposed to render viral proteins more susceptible to proteasomal degradation [4], and FAM46B was demonstrated to favour ubiquitination of β-catenin in BC cells [36]. How this effect enters the already intricate picture of FAM46C functions is hard to define and will for sure be the focus of future studies. What is instead clearly defined is that the intracellular pathways affected by FAM46C might be broader than one might think.

Future studies should explore the link between the functional models proposed and the intracellular pathways modulated by FAM46C.

In general, having a more exhaustive picture of FAM46C activities and of how FAM46C is modulating intracellular pathways in specific cell types will also help us shed more light on the synergistic effect of FAM46C expression/down-modulation with drug sensitivity.

In this same direction, it will be important to define how *FAM46C* gene expression is actually being regulated in different environments, as *FAM46C* seems to be activated downstream of quite different stimuli in different cell types. In this direction, a thorough exploratory study to define regulatory elements throughout *FAM46C* gene would be highly informative.

Knowing how *FAM46C* expression is modulated will be really important to actively predict/control its tumour suppressor effects. Specifically, if *FAM46C* can be induced downstream of specific receptor engagement, as demonstrated by Bilska and colleagues [71], it would be tempting to speculate to improve cancer patient therapy by up-modulating FAM46C protein levels through simple receptor activation.

Although the relevance of FAM46C in cancer is undeniable, much work still has to be carried out to better define how, when, and where FAM46C is exerting its functions, in order to exploit this information for implementing cancer patient treatment.

## Figures and Tables

**Table 1:** List of studies regarding FAM46C role in different cancers.

References #	Cancer Type	Cell Line/Cell Type/Sample	Relevant FAM46C-Related Phenotype
[7]	HCC	SMMC-7721, MHCC-97H, SK-Hep-1	Effect on cell proliferation/apoptosis
[8]	MM	OCIMY5	Effect on cell growth/apoptosis
[9]	CRC	Primary Tumour	Reduced levels in tumour vs. control samples
[10]	PC	DU145	Effect on cell growth/apoptosis
[11]	GC	MKN45, MKN74, AGS	Effect on cell proliferation/cell cycle/apoptosis
[12]	Oral SCC	HSC4, SCC15, and CAL27	Effect on cell proliferation/apoptosis
[13]	MM	KMS-11, OCI-My5, ANBL-6	Effect on tumour growth
[14]	HCC	SMMC-7721, MHCC-97H	Effect on cell migration/invasion
[20]	MM	SKMM1, H929	Effect on cell growth/survival
[21]	MM	LP-1, OPM-2	Effect on cell proliferation/cell cycle/apoptosis/tumour progression
[30]	MM	JJN3, RPMI-8226	Effect on cell migration/invasion
[57]	GC	MKN1, MKN45, MKN74, NUGC2, NUGC3, NUGC4, SC-6-JCK, AGS, KATOIII, N87, GCIY, patients tissues	Reduced levels in tumour cell lines/samples vs. controls
[60]	CRC	Tumour	Reduced levels in tumour vs. control samples
[61]	Lung SCC	PC-10, Tumour	Effect on cell proliferation and reduced levels in tumour vs. control samples
[62]	Oesophageal SCC	KYSE30 and TE-13	Effect on cell proliferation/migration/invasion
[63]	LC	H292, A549	Putative effect on cell proliferation/migration
[64]	MM	MM.1S	Effect on cell viability/proliferation
[65]	MM	RPMI-8226	Effect on cell apoptosis
[66]	MM	RPMI-8226, OPM-2, MM.1S, U266	Effect on clonogenic potential/proliferation/apoptosis
[67]	OS	MG-63	Effect on cell growth/migration/invasion
[68]	MM	Patient	Expression predicts extramedullary metastasis
[69]	MM	CD138^+^ BM mice cells	Expression correlates with increased survival

For each paper listed, we have summarized the tumour type, cell line/cell type/sample and relevant FAM46C-related phenotype analysed. Abbreviations: #: number; CRC: colorectal cancer; GC: gastric cancer; HCC: hepatocellular carcinoma; LC: lung cancer; MM: multiple myeloma; OS: osteosarcoma; PC: prostate cancer; SCC: squamous cell carcinoma.

**Table 2:** Most relevant studies regarding FAM46C involvement in intracellular pathway regulation.

References #	Cell Type/Cancer Type	Cell Line	Pathway Involved	Effect Related to FAM46C Expression
[7]	HCC	SMMC-7721, MHCC-97H, SK-Hep-1	MAPK/ERK	Inhibition
[8]	MM	OCIMY5, XG1	MAPK/ERK	Inhibition
[10]	PC	DU145	PI3K/AKT/mTOR	Inhibition through PTEN stabilization
[11]	GC	MKN45, MKN74, AGS	WNT/Beta-catenin	Inhibition
[12]	Oral SCC	HSC4, SCC15, CAL27	MAPK/ERK	Inhibition
[13]	MM	KMS-11, OCI-My5, ANBL-6	PI3K/AKT/mTOR	Inhibition
[14]	HCC	SMMC-7721; MHCC-97H	TGF-beta/SMAD	Inhibition
[70]	Cardiomyocyte	AC16	MAPK/ERK	Inhibition

For each paper, we have listed the cell type/tumour type and cell line analysed and the pathway regulated by FAM46C, underlining its effects. Abbreviations: #: number; GC: gastric cancer; HCC: hepatocellular carcinoma; MM: multiple myeloma; PC: prostate cancer; SCC: squamous cell carcinoma.

**Table 3:** Most relevant studies regarding FAM46C functional models.

References #	Cell Type/Cancer Type	Cell Line	Associated Functional Model	Effect
[4]	Epithelial	HEK-293T	Intracellular trafficking regulation	Inhibition of lentiviral particle production
[9]	OS, M	U2OS, MDA-MB-435	Inhibition of PLK4	Inhibition of cell invasion/cancer growth
[17]	Epithelial, murine osteoblast	HEK-293T	Poly(A) polymerase	Proper bone formation
[20]	Epithelial, MM	HEK-293, SKMM1, H929	Poly(A) polymerase	Stabilization of ER-targeted proteins
[21]	MM	LP-1, RPMI-8226	Intracellular trafficking regulation	Protein aggregate accumulation and consequent apoptosis induction
[22]	-	-	Poly(A) polymerase	-
[27]	Murine macrophage	-	Poly(A) polymerase	Innate immune response regulation
[30]	MM	U266, JJN3, RPMI-8226	Poly(A) polymerase	Increased production of Ig light chains and BIP protein
[64]	MM	MM1.S	Poly(A) polymerase	Inhibition of cell viability/proliferation
[65]	MM	RPMI-8226	Poly(A) polymerase	Polyadenylation of RNA molecules with poly(A) tails
[71]	Murine spleen-derived B cells	-	Poly(A) polymerase	Ig mRNA stabilization and enhanced expression

For each paper listed, we have summarized the cell type/cancer type, cell lines analysed, and the connection with one of the specific functional models proposed. Abbreviations: #: number; MM: multiple myeloma.

**Table 4:** Most relevant studies regarding regulation of *FAM46C* expression.

References #	Cell Type/Cancer Type	Cell Line	Regulator of Fam46c Expression	Effect on FAM46C Expression
[4]	Macrophage/Dendritic cell/CD4^+^ T cell	Primary	IFN-α, IFN-γ	Stimulation
[18]	Macrophage	THP-1	miR-657	Inhibition
[19]	Macrophage	Raw264.7	circRNA_17725, miR-4668-5p	circRNA_17725 sponges miR-4668-5p, upregulating FAM46C
[61]	Lung SCC	PC10, ACC-LC-73	miR-296-5p, miR-324-3p, miR-3928-3p	Inhibition
[62]	Oesophageal SCC	KYSE30, TE-13	miR-1269a	Inhibition
[63]	LC	H292, A549	HIC1	Stimulation (putative)
[66]	MM	RPMI-8226, U266	PRDM1	Stimulation
[67]	OS	MG-63	miR10-b	Inhibition
[71]	Murine naïve B	Primary	LPS/IL-4 or engagement of TLR1/2, TLR2, TLR4, TLR6/2, TLR9	Stimulation
[86]	MM	Primary bone marrow plasma cells	MSTRG.13132	Correlation

For each paper listed, we have summarized the cell type/tumour type and cell line analysed and the regulator(s) of FAM46C expression. Abbreviations: #: number; LC: lung cancer; MM: multiple myeloma; OS: osteosarcoma; SCC: squamous cell carcinoma.

**Table 5:** Most relevant studies regarding FAM46C synergistic effects on anti-cancer drug treatment.

References #	Cancer Type	Cell Line/Model	Drug Tested	Effect of FAM46C
[7]	HCC	SMMC-7721, MHCC-97H, SK-Hep-1	NTCD	Involvement in drug-induced anti-proliferative and anti-metastatic effects
[8]	MM	OCIMY5	BTZ, LENA	Depletion confers resistance to DEXA but not to BTZ
[8]	MM	XG1	DEXA, LENA	Depletion confers resistance to both DEXA and LENA
[10]	PC	DU145, 22RV1	DTX	Expression increases while down-modulation decreases sensitivity
[10]	PC	Patient-derived Xenograft	DTX	Higher expression increases sensitivity
[10]	PC	Primary human cancer cells	DTX	Higher expression increases sensitivity
[13]	MM	KMS-11	BTZ, LENA, POMA, DOXO, MEL	Knockout confers resistance to DOXO or MEL administration
[13]	MM	ANBL-6	BTZ, LENA, POMA	Knockout is synergistic with BTZ or POMA administration
[13]	MM	OCI-My5	DOXO, MEL	Knockout confers resistance to DOXO or MEL administration
[14]	HCC	SMMC-7721, MHCC-97H	NTCD	Involvement in drug-induced anti-proliferative and anti-metastatic effects
[21]	MM	LP-1, OPM-2, U266	SK1-I	Expression increases while down-modulation decreases sensitivity
[30]	MM	RPMI-8226	BTZ, DEXA, MEL	None
[30]	MM	JJN3	BTZ, MEL	None
[68]	MM	Patient-derived Bone Marrow cells	BTZ	Low levels are synergistic with BTZ administration
[68]	MM	Patient-derived Bone Marrow cells	DEXA	High levels are synergistic with DEXA administration
[69]	MM	CD138^+^ cells derived from VQ mice models	BTZ	Reduced levels confer resistance

For each paper listed, we have summarized the tumour type and cell lines analysed and the drugs tested for synergistic effects with FAM46C expression/down-modulation. Abbreviations: #: number; BTZ: bortezomib; DTX: docetaxel; DEXA: dexamethasone; LENA: lenalidomide; MEL: melphalan; POMA: pomalidomide; HCC: hepatocellular carcinoma; MM: multiple myeloma; PC: prostate cancer.
